# *catena*-Poly[[bis­[2-(2,3-dimethyl­anilino)benzoato-κ*O*]cadmium(II)]-di-μ-3-pyridylmethanol-κ^2^ *N*:*O*;κ^2^ *O*:*N*]

**DOI:** 10.1107/S1600536808003000

**Published:** 2008-02-06

**Authors:** Jan Moncol, Dušan Mikloš, Peter Segľa, Marian Koman, Tadeusz Lis

**Affiliations:** a Department of Inorganic Chemistry, Slovak, Technical University, Radlinského 9, SK-812 37 Bratislava, Slovakia; b Faculty of Chemistry, University of Wrocław, 14 Joliot-Curie St, 50-383 Wrocław, Poland

## Abstract

In the crystal structure of the title compound, [Cd(C_15_H_14_NO_2_)_2_(C_6_H_7_NO)_2_]_*n*_, the Cd atom displays a distorted octa­hedral geometry, including two pyridine N atoms and two hydroxyl O from four symmetry-related 3-pyridylmethanol (3-pyme) ligands and two carboxylate O atoms from mefenamate [2-(2,3-dimethyl­anilino)benzoate] anions. The Cd atoms are connected *via* the bridging 3-pyme ligands into chains, that extend in the *a-*axis direction. The Cd atom is located on a center of inversion, whereas the 3-pyme ligands and the mefenamate anions occupy general positions.

## Related literature

For related literature, see: Cini (2000 ▶); Lörinc *et al.* (2004 ▶); Moncol *et al.* (2006 ▶); Valach *et al.* (1997 ▶); Weder *et al.* (2002 ▶).
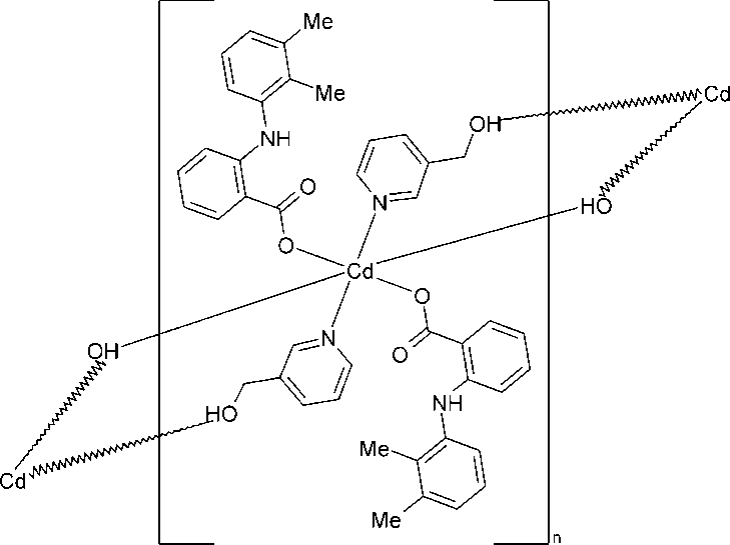


## Experimental

### 

#### Crystal data

[Cd(C_15_H_14_NO_2_)_2_(C_6_H_7_NO)_2_]
*M* *_r_* = 811.20
Triclinic, 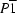

*a* = 6.829 (2) Å
*b* = 7.765 (2) Å
*c* = 16.930 (4) Å
α = 79.25 (3)°
β = 85.31 (3)°
γ = 86.71 (3)°
*V* = 878.2 (4) Å^3^
*Z* = 1
Mo *K*α radiation
μ = 0.68 mm^−1^
*T* = 100 (2) K
0.15 × 0.08 × 0.02 mm

#### Data collection

Kuma KM-4 CCD diffractometer
Absorption correction: analytical (Clark & Reid, 1995 ▶) *T*_min_ = 0.915, *T*_max_ = 0.985
16779 measured reflections
6275 independent reflections
5024 reflections with *I* > 2σ(*I*)
*R*_int_ = 0.063

#### Refinement

*R*[*F* ^2^ > 2σ(*F* ^2^)] = 0.062
*wR*(*F* ^2^) = 0.087
*S* = 1.01
6275 reflections
243 parameters
H-atom parameters constrained
Δρ_max_ = 1.29 e Å^−3^
Δρ_min_ = −0.99 e Å^−3^

### 

Data collection: *CrysAlis CCD* (Oxford Diffraction, 2006 ▶); cell refinement: *CrysAlis RED* (Oxford Diffraction, 2006 ▶); data reduction: *CrysAlis RED*; program(s) used to solve structure: *SHELXS97* (Sheldrick, 2008 ▶); program(s) used to refine structure: *SHELXL97* (Sheldrick, 2008 ▶); molecular graphics: *XP* in *SHELXTL* (Sheldrick, 2008 ▶); software used to prepare material for publication: *enCIFer* (Allen *et al.*, 2004 ▶).

## Supplementary Material

Crystal structure: contains datablocks global, I. DOI: 10.1107/S1600536808003000/nc2089sup1.cif

Structure factors: contains datablocks I. DOI: 10.1107/S1600536808003000/nc2089Isup2.hkl

Additional supplementary materials:  crystallographic information; 3D view; checkCIF report

## Figures and Tables

**Table:** 

Cd—O2	2.2475 (17)
Cd—O3^i^	2.3400 (17)
Cd—N2	2.348 (2)

**Table:** 

O2—Cd—O3^i^	91.10 (6)
O2—Cd—N2	89.76 (7)
O3^i^—Cd—N2	94.51 (7)

Symmetry code: (i) 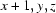.

**Table 2:** Hydrogen-bond geometry (Å, °)

*D*—H⋯*A*	*D*—H	H⋯*A*	*D*⋯*A*	*D*—H⋯*A*
N1—H1*N*⋯O1	0.91	1.97	2.696 (3)	135
O3—H3*O*⋯O1^ii^	0.84	1.78	2.600 (2)	164

Symmetry code: (ii) 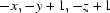.

**Fig. 1.**
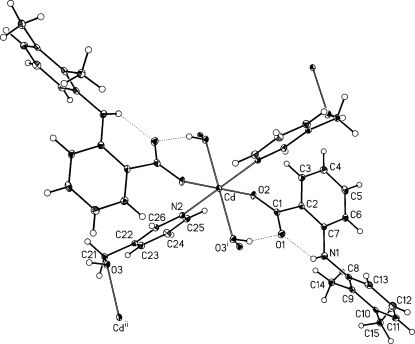
Perspective view of (I), with the atom numbering scheme and thermal ellipsoids drawn at the 30% probability level. Symmetry codes: i = x + 1, y, z, ii = x - 1, y, z.

**Fig. 2.**
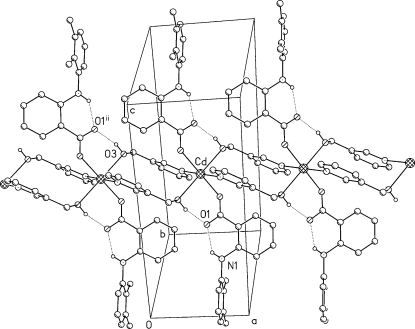
The crystal packing of the title compound viewed along the b axis. Symmetry codes: ii = x - 1, y, z.

## supplementary crystallographic information

### Comment

The importance of metal complexes with non-steroidal anti-inflammatory drugs (NSAIDs) as ligands has been stressed in several review papers (Cini, 2000; Weder *et al.*, 2002). One of these NSAIDs are fenamates, which are derivatives of *N*-phenylantranilic acid and 2-phenylaminonicotinic acid (mefenamic acid, niflumic acid, tolfenamic acid, flufenamic acid). As part of our efforts to investigate metal(II) complexes based on fenamates, we describe the X-ray characterization of the title compound.

The Cd^II^ atom are in a distorted octahedral coordination formed by two carboxyl O atoms of mefenamate anions [Cd–O2 = 2.2475 (17) Å], two pyridine N atoms of 3-pyridylmethanol ligands (3-pyme) [Cd–N2 = 2.348 (2) Å] and two hydroxyl O atoms of adjacent 3-pyridylmethanol ligands [Cd–O3 = 2.3400 (17) Å] /Fig. 1). Each of the Cd atoms is connected *via* two symmetry related 3-pyridylmethanol into chains, that elongated in the direction of the crystallographic *a* axis.

The uncoordinated O atom of the carboxylate group of the mefenamate anion forms an intramolecular N–H···O hydrogen bonds to the amine H atoms as well as as intermolecular O–H···O hydrogen bonds to the hydroxyl H atoms of the 3-pyridylmethanol ligand, creating six-membered rings (Table 2).

The crystal structure of (I) can be compared with those of polymeric copper(II) carboxylate complexes [Cu(RCO_2_)_2_(3-pyme)_2_]_n_, which forms either coordination polymers with two bridging 3-pyme ligands between two Cu^II^ atoms or two-dimensional coordination polymer with only one bridging 3-pyme ligand between two Cu^II^ atoms (Moncol *et al.*, 2006). [Cu(niflumate)_2_(3-pyme)_2_]_n_ for example is one-dimensional coordination polymer, (Valach *et al.*, 1997), while [Cu(flufenamate)_2_(3-pyme)_2_]_n_ is two-dimensional coordination polymer (Lörinc *et al.*, 2004).

### Experimental

The title complex was prepared by adding 3-pyridylmethanol (5 cm^3^) to a solution of Cd(mefenamato)_2_.H_2_O (1.25 mmol) in methanol (20 cm^3^). The fine white microcrystals which are formed by slow evaporation of the solvent on standing for a few days at room temperature were separated, washed with ethanol and dried *in vacuo*, yield 85%. The Anal. Calc.: C, 62.18; H, 5.22; N, 6.91; Cd, 13.86; Found: C, 61.82; H, 5.31; N, 7.00; Cd, 13.45. IR (KBr) cm^-1^: 3279 ν(N–H); 1610 ν_as_(COO^-^); 1385 ν_s_(COO^-^); 1056 ν(C–O)_3-pyme_; 643 δ(py)_3-pyme_.

### Refinement

All H atoms were placed in calculated positions (O–H allowed to rotate but not to tip and later fixed at the optimized position) and were refined isotropic with *U*_iso_(H) = 1.2 *U*_iso_(parent atom) using a riding model with C–H = 0.95, 0.99 and 0.98 Å, O–H = 0.84 Å and (N–H = 0.91 Å, respectively.

### Crystal data

**Table:**

[Cd(C_15_H_14_NO_2_)_2_(C_6_H_7_NO)_2_]	*Z* = 1
*M**_r_* = 811.20	*F*_000_ = 418
Triclinic, *P*1	*D*_x_ = 1.534 Mg m^−^^3^
Hall symbol: -P 1	Mo *K*α radiation λ = 0.71073 Å
*a* = 6.829 (2) Å	Cell parameters from 9193 reflections
*b* = 7.765 (2) Å	θ = 4–32º
*c* = 16.930 (4) Å	µ = 0.68 mm^−^^1^
α = 79.25 (3)º	*T* = 100 (2) K
β = 85.31 (3)º	Plate, colourless
γ = 86.71 (3)º	0.15 × 0.08 × 0.02 mm
*V* = 878.2 (4) Å^3^	

### Data collection

**Table:**

Kuma KM-4 CCD diffractometer	6275 independent reflections
Radiation source: fine-focus sealed tube	5024 reflections with *I* > 2σ(*I*)
Monochromator: graphite	*R*_int_ = 0.063
*T* = 100(2) K	θ_max_ = 32.5º
ω scans	θ_min_ = 4.6º
Absorption correction: analytical(Clark & Reid, 1995)	*h* = −10→9
*T*_min_ = 0.915, *T*_max_ = 0.985	*k* = −11→11
16779 measured reflections	*l* = −23→25

### Refinement

**Table:**

Refinement on *F*^2^	Secondary atom site location: difference Fourier map
Least-squares matrix: full	Hydrogen site location: inferred from neighbouring sites
*R*[*F*^2^ > 2σ(*F*^2^)] = 0.062	H-atom parameters constrained
*wR*(*F*^2^) = 0.087	*w* = 1/[σ^2^(*F*_o_^2^) + (0.0274*P*)^2^] where *P* = (*F*_o_^2^ + 2*F*_c_^2^)/3
*S* = 1.01	(Δ/σ)_max_ < 0.001
6275 reflections	Δρ_max_ = 1.29 e Å^−^^3^
243 parameters	Δρ_min_ = −0.99 e Å^−^^3^
Primary atom site location: structure-invariant direct methods	Extinction correction: none

### Special details

**Table:**

Experimental. face-indexed (*CrysAlis RED*; Oxford Diffraction, 2006)
Geometry. All e.s.d.'s (except the e.s.d. in the dihedral angle between two l.s. planes) are estimated using the full covariance matrix. The cell e.s.d.'s are taken into account individually in the estimation of e.s.d.'s in distances, angles and torsion angles; correlations between e.s.d.'s in cell parameters are only used when they are defined by crystal symmetry. An approximate (isotropic) treatment of cell e.s.d.'s is used for estimating e.s.d.'s involving l.s. planes.
Refinement. Refinement of *F*^2^ against ALL reflections. The weighted *R*-factor *wR* and goodness of fit *S* are based on *F*^2^, conventional *R*-factors *R* are based on *F*, with *F* set to zero for negative *F*^2^. The threshold expression of *F*^2^ > σ(*F*^2^) is used only for calculating *R*-factors(gt) *etc*. and is not relevant to the choice of reflections for refinement. *R*-factors based on *F*^2^ are statistically about twice as large as those based on *F*, and *R*- factors based on ALL data will be even larger.

### Fractional atomic coordinates and isotropic or equivalent isotropic displacement parameters (Å^2^)

**Table:**

	*x*	*y*	*z*	*U*_iso_*/*U*_eq_	
Cd	0.5000	0.5000	0.5000	0.01174 (8)	
N1	0.7197 (3)	0.2022 (3)	0.20406 (12)	0.0165 (5)	
N2	0.2681 (3)	0.7076 (2)	0.43955 (11)	0.0116 (4)	
O1	0.5810 (2)	0.2645 (2)	0.35027 (10)	0.0179 (4)	
O2	0.6740 (2)	0.5163 (2)	0.38047 (9)	0.0142 (4)	
O3	−0.3074 (2)	0.7089 (2)	0.53597 (9)	0.0139 (4)	
C1	0.6931 (3)	0.3927 (3)	0.34046 (14)	0.0130 (5)	
C2	0.8654 (3)	0.3967 (3)	0.27859 (14)	0.0116 (5)	
C3	1.0243 (3)	0.4955 (3)	0.28732 (15)	0.0158 (5)	
H3	1.0159	0.5627	0.3290	0.019*	
C4	1.1945 (4)	0.4974 (3)	0.23628 (15)	0.0176 (5)	
H4	1.3022	0.5640	0.2433	0.021*	
C5	1.2045 (4)	0.4011 (3)	0.17537 (15)	0.0178 (6)	
H5	1.3208	0.4008	0.1405	0.021*	
C6	1.0488 (3)	0.3050 (3)	0.16404 (14)	0.0148 (5)	
H6	1.0583	0.2418	0.1209	0.018*	
C7	0.8761 (3)	0.2994 (3)	0.21548 (14)	0.0127 (5)	
C8	0.7302 (3)	0.0801 (3)	0.14937 (15)	0.0151 (5)	
C9	0.7325 (3)	0.1415 (3)	0.06625 (15)	0.0154 (5)	
C10	0.7413 (3)	0.0186 (3)	0.01387 (15)	0.0144 (5)	
C11	0.7457 (3)	−0.1599 (3)	0.04723 (15)	0.0175 (5)	
H11	0.7522	−0.2432	0.0124	0.021*	
C12	0.7409 (4)	−0.2189 (3)	0.12952 (16)	0.0193 (6)	
H12	0.7423	−0.3411	0.1507	0.023*	
C13	0.7341 (3)	−0.0990 (3)	0.18106 (15)	0.0175 (5)	
H13	0.7321	−0.1388	0.2377	0.021*	
C14	0.7222 (4)	0.3341 (3)	0.03169 (15)	0.0175 (5)	
H14A	0.6992	0.4009	0.0756	0.026*	
H14B	0.6142	0.3600	−0.0040	0.026*	
H14C	0.8465	0.3673	0.0010	0.026*	
C15	0.7481 (4)	0.0776 (4)	−0.07602 (15)	0.0188 (6)	
H15A	0.8841	0.0999	−0.0970	0.028*	
H15B	0.6663	0.1855	−0.0890	0.028*	
H15C	0.6983	−0.0141	−0.1007	0.028*	
C21	−0.2568 (3)	0.8672 (3)	0.48322 (15)	0.0130 (5)	
H21A	−0.2578	0.9633	0.5144	0.016*	
H21B	−0.3555	0.8987	0.4426	0.016*	
C22	−0.0558 (3)	0.8469 (3)	0.44113 (14)	0.0119 (5)	
C23	−0.0057 (4)	0.9524 (3)	0.36745 (15)	0.0164 (5)	
H23	−0.0984	1.0372	0.3428	0.020*	
C24	0.1808 (3)	0.9330 (3)	0.33008 (15)	0.0176 (5)	
H24	0.2169	1.0035	0.2794	0.021*	
C25	0.3126 (3)	0.8097 (3)	0.36774 (14)	0.0145 (5)	
H25	0.4399	0.7964	0.3419	0.017*	
C26	0.0861 (3)	0.7263 (3)	0.47475 (14)	0.0116 (5)	
H26	0.0531	0.6532	0.5251	0.014*	
H1N	0.6340	0.1764	0.2482	0.014*	
H3O	−0.3791	0.7267	0.5766	0.014*	

### Atomic displacement parameters (Å^2^)

**Table:**

	*U*^11^	*U*^22^	*U*^33^	*U*^12^	*U*^13^	*U*^23^
Cd	0.01022 (13)	0.01148 (13)	0.01335 (14)	0.00098 (10)	0.00044 (9)	−0.00282 (10)
N1	0.0151 (10)	0.0236 (12)	0.0118 (11)	−0.0023 (9)	0.0033 (8)	−0.0071 (9)
N2	0.0111 (9)	0.0112 (10)	0.0129 (10)	−0.0010 (8)	0.0005 (8)	−0.0036 (8)
O1	0.0177 (9)	0.0199 (9)	0.0169 (9)	−0.0040 (8)	0.0039 (7)	−0.0068 (8)
O2	0.0170 (8)	0.0127 (8)	0.0124 (9)	0.0014 (7)	0.0046 (7)	−0.0041 (7)
O3	0.0138 (8)	0.0140 (9)	0.0142 (9)	−0.0035 (7)	0.0045 (7)	−0.0045 (7)
C1	0.0142 (11)	0.0138 (12)	0.0097 (12)	0.0040 (10)	−0.0026 (9)	0.0003 (10)
C2	0.0127 (11)	0.0110 (11)	0.0095 (11)	0.0022 (9)	0.0006 (9)	0.0008 (9)
C3	0.0185 (12)	0.0152 (12)	0.0136 (13)	0.0013 (10)	−0.0017 (10)	−0.0025 (10)
C4	0.0165 (12)	0.0196 (13)	0.0164 (13)	−0.0033 (10)	0.0010 (10)	−0.0027 (11)
C5	0.0140 (12)	0.0219 (14)	0.0143 (13)	0.0017 (10)	0.0061 (10)	0.0007 (11)
C6	0.0171 (12)	0.0165 (12)	0.0101 (12)	0.0041 (10)	0.0000 (9)	−0.0031 (10)
C7	0.0134 (11)	0.0116 (12)	0.0126 (12)	0.0008 (9)	−0.0007 (9)	−0.0017 (10)
C8	0.0089 (11)	0.0211 (13)	0.0154 (13)	0.0000 (10)	0.0009 (9)	−0.0048 (11)
C9	0.0095 (11)	0.0168 (13)	0.0192 (13)	0.0011 (9)	0.0003 (9)	−0.0025 (11)
C10	0.0071 (11)	0.0196 (13)	0.0167 (13)	0.0022 (9)	−0.0010 (9)	−0.0044 (11)
C11	0.0141 (12)	0.0232 (14)	0.0171 (13)	−0.0024 (10)	−0.0001 (10)	−0.0084 (11)
C12	0.0179 (13)	0.0156 (13)	0.0236 (14)	−0.0018 (10)	−0.0015 (11)	−0.0009 (11)
C13	0.0159 (12)	0.0208 (14)	0.0155 (13)	−0.0022 (10)	−0.0012 (10)	−0.0023 (11)
C14	0.0191 (13)	0.0173 (13)	0.0162 (13)	0.0013 (10)	−0.0006 (10)	−0.0042 (11)
C15	0.0166 (12)	0.0224 (14)	0.0176 (13)	0.0029 (11)	0.0003 (10)	−0.0055 (11)
C21	0.0113 (11)	0.0102 (11)	0.0172 (13)	0.0003 (9)	−0.0009 (9)	−0.0023 (10)
C22	0.0116 (11)	0.0108 (11)	0.0143 (12)	−0.0020 (9)	−0.0006 (9)	−0.0045 (10)
C23	0.0168 (12)	0.0144 (12)	0.0174 (13)	0.0011 (10)	−0.0033 (10)	−0.0009 (11)
C24	0.0177 (12)	0.0189 (13)	0.0135 (13)	−0.0018 (10)	0.0006 (10)	0.0032 (11)
C25	0.0147 (12)	0.0163 (13)	0.0124 (12)	−0.0036 (10)	0.0032 (9)	−0.0030 (10)
C26	0.0133 (11)	0.0116 (12)	0.0094 (11)	−0.0019 (9)	0.0024 (9)	−0.0018 (9)

### Geometric parameters (Å, °)

**Table:**

Cd—O2^i^	2.2475 (17)	C8—C9	1.398 (3)
Cd—O2	2.2475 (17)	C9—C10	1.415 (3)
Cd—O3^ii^	2.3400 (17)	C9—C14	1.501 (3)
Cd—O3^iii^	2.3400 (17)	C10—C11	1.396 (3)
Cd—N2	2.348 (2)	C10—C15	1.503 (3)
Cd—N2^i^	2.348 (2)	C11—C12	1.381 (3)
N1—C7	1.389 (3)	C11—H11	0.9500
N1—C8	1.439 (3)	C12—C13	1.386 (4)
N1—H1N	0.91	C12—H12	0.9500
N2—C25	1.344 (3)	C13—H13	0.9500
N2—C26	1.346 (3)	C14—H14A	0.9800
O1—C1	1.269 (3)	C14—H14B	0.9800
O2—C1	1.269 (3)	C14—H14C	0.9800
O3—C21	1.420 (3)	C15—H15A	0.9800
O3—Cd^iv^	2.3400 (17)	C15—H15B	0.9800
O3—H3O	0.84	C15—H15C	0.9800
C1—C2	1.507 (3)	C21—C22	1.508 (3)
C2—C3	1.397 (3)	C21—H21A	0.9900
C2—C7	1.414 (3)	C21—H21B	0.9900
C3—C4	1.389 (3)	C22—C23	1.387 (3)
C3—H3	0.9500	C22—C26	1.388 (3)
C4—C5	1.378 (4)	C23—C24	1.388 (3)
C4—H4	0.9500	C23—H23	0.9500
C5—C6	1.379 (3)	C24—C25	1.379 (3)
C5—H5	0.9500	C24—H24	0.9500
C6—C7	1.405 (3)	C25—H25	0.9500
C6—H6	0.9500	C26—H26	0.9500
C8—C13	1.394 (3)		
			
O2^i^—Cd—O2	180.000 (1)	C8—C9—C10	119.0 (2)
O2^i^—Cd—O3^ii^	91.10 (6)	C8—C9—C14	121.6 (2)
O2—Cd—O3^ii^	88.90 (6)	C10—C9—C14	119.5 (2)
O2^i^—Cd—O3^iii^	88.90 (6)	C11—C10—C9	118.7 (2)
O2—Cd—O3^iii^	91.10 (6)	C11—C10—C15	120.2 (2)
O3^ii^—Cd—O3^iii^	180.00 (5)	C9—C10—C15	121.1 (2)
O2^i^—Cd—N2	90.24 (7)	C12—C11—C10	121.8 (2)
O2—Cd—N2	89.76 (7)	C12—C11—H11	119.1
O3^ii^—Cd—N2	85.49 (7)	C10—C11—H11	119.1
O3^iii^—Cd—N2	94.51 (7)	C11—C12—C13	119.8 (2)
O2^i^—Cd—N2^i^	89.76 (7)	C11—C12—H12	120.1
O2—Cd—N2^i^	90.24 (7)	C13—C12—H12	120.1
O3^ii^—Cd—N2^i^	94.51 (7)	C12—C13—C8	119.6 (2)
O3^iii^—Cd—N2^i^	85.49 (7)	C12—C13—H13	120.2
N2—Cd—N2^i^	180.00 (9)	C8—C13—H13	120.2
C7—N1—C8	124.02 (19)	C9—C14—H14A	109.5
C7—N1—H1N	114.1	C9—C14—H14B	109.5
C8—N1—H1N	115.4	H14A—C14—H14B	109.5
C25—N2—C26	118.0 (2)	C9—C14—H14C	109.5
C25—N2—Cd	120.73 (15)	H14A—C14—H14C	109.5
C26—N2—Cd	121.31 (15)	H14B—C14—H14C	109.5
C1—O2—Cd	124.12 (15)	C10—C15—H15A	109.5
C21—O3—Cd^iv^	123.21 (14)	C10—C15—H15B	109.5
C21—O3—H3O	112.5	H15A—C15—H15B	109.5
Cd^iv^—O3—H3O	96.9	C10—C15—H15C	109.5
O2—C1—O1	124.7 (2)	H15A—C15—H15C	109.5
O2—C1—C2	117.3 (2)	H15B—C15—H15C	109.5
O1—C1—C2	117.9 (2)	O3—C21—C22	110.59 (18)
C3—C2—C7	119.4 (2)	O3—C21—H21A	109.5
C3—C2—C1	117.5 (2)	C22—C21—H21A	109.5
C7—C2—C1	123.1 (2)	O3—C21—H21B	109.5
C4—C3—C2	121.4 (2)	C22—C21—H21B	109.5
C4—C3—H3	119.3	H21A—C21—H21B	108.1
C2—C3—H3	119.3	C23—C22—C26	117.8 (2)
C5—C4—C3	118.9 (2)	C23—C22—C21	120.4 (2)
C5—C4—H4	120.6	C26—C22—C21	121.9 (2)
C3—C4—H4	120.6	C22—C23—C24	119.5 (2)
C4—C5—C6	121.2 (2)	C22—C23—H23	120.2
C4—C5—H5	119.4	C24—C23—H23	120.2
C6—C5—H5	119.4	C25—C24—C23	118.9 (2)
C5—C6—C7	120.9 (2)	C25—C24—H24	120.6
C5—C6—H6	119.5	C23—C24—H24	120.6
C7—C6—H6	119.5	N2—C25—C24	122.6 (2)
N1—C7—C6	120.8 (2)	N2—C25—H25	118.7
N1—C7—C2	121.0 (2)	C24—C25—H25	118.7
C6—C7—C2	118.2 (2)	N2—C26—C22	123.3 (2)
C13—C8—C9	121.2 (2)	N2—C26—H26	118.4
C13—C8—N1	118.7 (2)	C22—C26—H26	118.4
C9—C8—N1	120.1 (2)		
			
O2^i^—Cd—N2—C25	162.39 (18)	C1—C2—C7—C6	−176.3 (2)
O2—Cd—N2—C25	−17.61 (18)	C7—N1—C8—C13	106.9 (3)
O3^ii^—Cd—N2—C25	−106.52 (18)	C7—N1—C8—C9	−74.3 (3)
O3^iii^—Cd—N2—C25	73.48 (18)	C13—C8—C9—C10	−0.7 (3)
O2^i^—Cd—N2—C26	−18.75 (17)	N1—C8—C9—C10	−179.5 (2)
O2—Cd—N2—C26	161.25 (17)	C13—C8—C9—C14	178.1 (2)
O3^ii^—Cd—N2—C26	72.33 (17)	N1—C8—C9—C14	−0.7 (3)
O3^iii^—Cd—N2—C26	−107.67 (17)	C8—C9—C10—C11	0.5 (3)
O3^ii^—Cd—O2—C1	−26.22 (17)	C14—C9—C10—C11	−178.4 (2)
O3^iii^—Cd—O2—C1	153.78 (17)	C8—C9—C10—C15	−178.8 (2)
N2—Cd—O2—C1	−111.71 (18)	C14—C9—C10—C15	2.3 (3)
N2^i^—Cd—O2—C1	68.29 (18)	C9—C10—C11—C12	0.3 (4)
Cd—O2—C1—O1	19.1 (3)	C15—C10—C11—C12	179.6 (2)
Cd—O2—C1—C2	−159.04 (14)	C10—C11—C12—C13	−0.9 (4)
O2—C1—C2—C3	21.1 (3)	C11—C12—C13—C8	0.6 (4)
O1—C1—C2—C3	−157.2 (2)	C9—C8—C13—C12	0.1 (4)
O2—C1—C2—C7	−162.0 (2)	N1—C8—C13—C12	178.9 (2)
O1—C1—C2—C7	19.8 (3)	Cd^iv^—O3—C21—C22	−96.45 (19)
C7—C2—C3—C4	−1.3 (4)	O3—C21—C22—C23	155.3 (2)
C1—C2—C3—C4	175.7 (2)	O3—C21—C22—C26	−26.0 (3)
C2—C3—C4—C5	0.8 (4)	C26—C22—C23—C24	0.6 (4)
C3—C4—C5—C6	0.6 (4)	C21—C22—C23—C24	179.3 (2)
C4—C5—C6—C7	−1.4 (4)	C22—C23—C24—C25	−0.6 (4)
C8—N1—C7—C6	10.3 (3)	C26—N2—C25—C24	1.0 (4)
C8—N1—C7—C2	−170.3 (2)	Cd—N2—C25—C24	179.88 (18)
C5—C6—C7—N1	−179.8 (2)	C23—C24—C25—N2	−0.2 (4)
C5—C6—C7—C2	0.8 (3)	C25—N2—C26—C22	−1.0 (3)
C3—C2—C7—N1	−178.9 (2)	Cd—N2—C26—C22	−179.84 (17)
C1—C2—C7—N1	4.2 (3)	C23—C22—C26—N2	0.2 (4)
C3—C2—C7—C6	0.6 (3)	C21—C22—C26—N2	−178.5 (2)

Symmetry codes: (i) −*x*+1, −*y*+1, −*z*+1; (ii) −*x*, −*y*+1, −*z*+1; (iii) *x*+1, *y*, *z*; (iv) *x*−1, *y*, *z*.

### Hydrogen-bond geometry (Å, °)

**Table:**

*D*—H···*A*	*D*—H	H···*A*	*D*···*A*	*D*—H···*A*
N1—H1N···O1	0.91	1.97	2.696 (3)	135
O3—H3O···O1^ii^	0.84	1.78	2.600 (2)	164

Symmetry codes: (ii) −*x*, −*y*+1, −*z*+1.
